# Itaconate as an immune modulator

**DOI:** 10.18632/aging.102057

**Published:** 2019-06-24

**Authors:** Anaísa V. Ferreira, Mihai G. Netea, Jorge Domínguez-Andrés

**Affiliations:** 1 Department of Internal Medicine and Radboud Center for Infectious diseases (RCI), Radboud University Nijmegen Medical Centre, 6500HB Nijmegen, the Netherlands; 2 Instituto de Ciências Biomédicas Abel Salazar (ICBAS), Universidade do Porto, Porto, 4050-313, Portugal; 3 Department for Genomics & Immunoregulation, Life and Medical Sciences Institute (LIMES), University of Bonn, Bonn, 53115, Germany; 4 Human Genomics Laboratory, Craiova University of Medicine and Pharmacy, Craiova, Romania

**Keywords:** itaconate, metabolism, immune system, sepsis, trained immunity

The immune system has been implicated in a myriad of different contexts, such as cancer, neurological disorders and aging. Tissue homeostasis is maintained through multiple regulatory mechanisms, among which the immune system. Deregulation of immune responses can in turn lead to tissue pathology and disease. Thus, understanding the mechanisms that regulate immune responses has been a focus of different fields within the scientific community. In recent years, an intriguing metabolite has been explored for its broad immunomodulatory properties, itaconate.

Itaconate has long been manufactured from the fungi *Aspergillus* species and used as a multi-functional industrial biomaterial. However, the last years have uncovered novel facets of itaconate in mammalian systems. Activated immune cells, chiefly of the myeloid lineage, have an increased expression of the immune-responsive gene 1 (IRG1) mitochondrial enzyme, which catalyses the decarboxylation of the Krebs cycle intermediate cis-aconitate to itaconate. On one hand, itaconate has direct antibacterial properties, since it can inhibit bacterial growth in glucose-poor conditions. Notably, some bacteria can degrade itaconate to pyruvate and acetyl-CoA and thus survive in the eukaryotic host. These detoxifying pathways could also confer advantage against itaconate-producing fungi. On the other hand, in inflammatory conditions, the rising levels of itaconate have an extremely relevant role in keeping in check the inflammatory response. Lipopolysaccharides and type I interferons induce increased IRG1 expression and the production of the latter is limited by itaconate, indicating a regulatory negative feedback loop [1].

The molecular mechanisms under control by itaconate vary, but the net function is considered to be anti-inflammatory. Itaconate acts as a competitive succinate dehydrogenase (SDH) inhibitor, impairing the electron transport chain (ETC) with a concomitant succinate accumulation [2]. SDH is both an enzyme of the Krebs cycle, oxidizing succinate to fumarate, and the component of complex II of the ETC, feeding electrons derived from succinate oxidation to the ubiquinone pool. Itaconate has also been shown to inhibit fructose-6-phosphate 2-kinase and thereby possibly blocks the increased glycolysis of pro-inflammatory macrophages. Itaconate reacts with thiols, forming adducts with glutathione, the major cellular antioxidant, and producing higher levels of reactive oxygen species (ROS) [3]. The antioxidant Nrf2 pathway is then activated in the presence of itaconate, possibly indirectly due to the increased ROS production, but also due to direct alkylation of cysteine residues of the Nrf2 inhibitor Keap1 [1]. Ultimately, there is enhanced expression of antioxidant and a decreased expression of pro-inflammatory genes, due to the induction of Nrf2 and the transcription factor ATF3 [3], respectively. Interestingly, it has been suggested that itaconate increases the level of MHC class I in macrophages. However, its influence on antigen presentation remains to be explored. In addition, itaconate could conceptually regulate gene expression through epigenetic alterations. It sequesters glutathione, thereby possibly influencing histone glutathionylation and the levels of the methylation substrate s-adenosyl methionine (SAM). Moreover, an intermediate of itaconate catabolism has been negatively implicated in vitamin B12 metabolism [4]. The reduced levels of B12 affects methionine and folate cycles, relevant for not only glutathione synthesis and redox homeostasis but also for SAM levels and histone and DNA methylation.

The immunosuppressive effects of itaconate have been explored in various disease models, such as cardiac ischemia reperfusion injury, psoriasis, cancer and infection. In *in vivo* mouse models, intravenous administration of itaconate reduces myocardial infarct size [2], while the induction of psoriasis with itaconate treatment prevented the typical scaling and edema of the skin [3]. In a peritoneal tumor mouse model, high itaconate levels in tumor associated macrophages promote tumor progression. Itaconate plays important roles during infection, decreasing tissue injury in a *Mycobacterium tuberculosis* infection mouse model [5] and increasing macrophage bactericidal activity in a zebrafish model. Interestingly, itaconate altered neuronal metabolism to suppress viral replication in a mouse model of Zika virus infection [6], indicating that the modulatory effects of itaconate are not curtailed to cells of the myeloid lineage.

Itaconate could also be a possible modulator of maladaptive immune programs. In sepsis, the exacerbated inflammatory response to infection is counteracted by the induction of tolerance, which can lead to immune paralysis and an increased susceptibility to secondary infections. Monocyte immune tolerance is promoted by itaconate, which decreases the production of pro-inflammatory mediators. Heme oxygenase 1, a cytoprotective enzyme that limits inflammation, is expressed during tolerance and has also been shown to regulate IRG1 expression. In contrast, the immune system can be hyperactivated. Particularly, the innate immune system can be primed to exhibit an increased responsiveness upon a second non-related insult, mirroring an immunologic memory which was coined trained immunity [7]. One stimulus that can exert trained immunity in monocytes is the *C. albicans* cell wall component β-glucan. It has been recently described by our group that β-glucan-induced training can prevent endotoxin tolerance [8]. Exposure to β-glucan inhibits the increased IRG1 expression and thus preserves a functional SDH pathway and the integrity of the Krebs cycle. Interestingly, exposure to itaconate can also inhibit the induction of β-glucan trained immunity. In this model, itaconate is also considered to have anti-inflammatory effects, regulating tolerance positively while decreasing trained immunity.

In conclusion, itaconate metabolism could be both a possible therapeutic target and a tool in inflammatory and autoimmune diseases with altered immune responses. Further exploration of the molecular, metabolic and epigenetic changes induced by itaconate in different cell types and disease models will be of great importance, in order to better establish the potential of the modulation of this promising metabolite and open new therapeutic avenues.
